# High-Resolution Electrospray and Ion Mobility Sequential Mass Spectrometry for Structural Characterisation of Anticancer Stem Cell Agent Salinomycin and Its Isomers

**DOI:** 10.3390/molecules30234512

**Published:** 2025-11-22

**Authors:** Candy Jiang, Paul J. Gates

**Affiliations:** School of Chemistry, University of Bristol, Cantock’s Close, Bristol BS8 1TS, UK; candy.jiang@bristol.ac.uk

**Keywords:** salinomycin, polyether ionophores, sequential mass spectrometry, ion mobility mass spectrometry

## Abstract

Salinomycin is a polyether ionophore natural product widely studied for its anticancer stem cell properties and well established anti-coccidial activity. However, its complex structure and tendency to isomerise in solution complicates its mass spectrometric analysis. In this study, a combination of high-resolution accurate mass electrospray sequential mass spectrometry, ion mobility spectrometry and computational modelling was employed to analyse salinomycin and its isomers for the first time. Product ions generated from salinomycin and its isomer in the MS^n^ analysis are distinguished, and detailed fragmentation mechanisms are proposed. The novel application of ion mobility mass spectrometry to separate isomeric salinomycins provides revolutionary insight into the chelation positions of sodium by salinomycin (‘ionoforms’). The cation position has a fundamental effect on the fragmentation routes observed. These observations were supported by Gaussian modelling and collision cross-section calculations. The relationship between collision energy and peak intensity of all identifiable forms of salinomycin and respective product ions was visualised by a 3D energy breakdown graph. Results from this study provided firm grounding for understanding complex structures such as salinomycin. The methodology demonstrated here could be applied to a wide range of natural products and in other drug development or metabolomic studies.

## 1. Introduction

**Figure 1:**
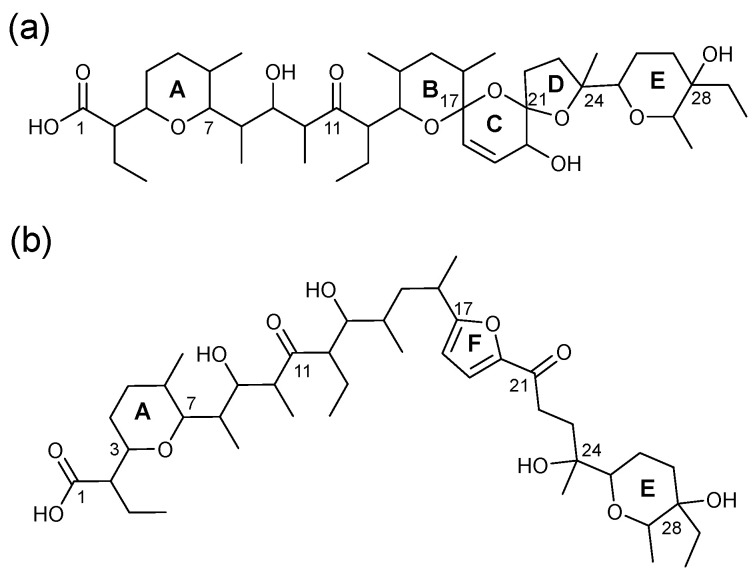
The structures (linear representation) of the natural (**a**) and isomeric (**b**) forms of salinomycin (formula: C_42_H_70_O_11_; Mw = 750). The bold letters **A**, **B**, **C**, **D**, **E** and **F** refer to the ether rings and are used throughout the discussion.

Coccidiosis is a parasitic disease of farmed animals kept in confinement and contaminated with coccidia oocysts [1,2]. This causes the destruction of the intestinal cells of the host animals, preventing nutrient absorbance and leading to haemorrhaging. In poultry farming, chickens affected with coccidiosis have lower egg production, weight loss, higher mortality rates and poorer meat quality [3]. Coccidiosis is also commonly observed in the farming of ruminants where loss of absorptive capacity of the gut leads to the lowering of the appetite and consequently reduced growth, diarrhoea, dehydration, abdominal pain and dysentery [4,5]. This disease results in suffering of the animals concerned and causes a high economic impact. Therefore, prevention is crucial for both animal welfare and economic reasons. Salinomycin (SAL) (see Figure 1), a polyether ionophore first reported in 1974, is a highly effective coccidiostat—a veterinary antibiotic drug used to treat coccidiosis—and used as a general feed additive to reduce disease occurrence. SAL functions by forming lipid-soluble complexes with alkali metal cations, causing disruption to the osmotic balance across the cell membrane, resulting in cell death [6].

In recent years, SAL has been found to reduce the proportion of breast cancer stem cells by 100-fold relative to a commonly used chemotherapeutic drug. The treatment of infected mice with SAL also showed in vivo inhibition in mammary tumour growth [7]. Since the publication of this study, an influx of research has been conducted on SAL, especially in its application in cancer treatment. The pathways of how SAL could kill cancer stem cells have been investigated [8,9], and it has been stated that the antitumour mechanism for SAL is connected to its strong affinity to potassium cations [10]. This important conclusion reflects an earlier study on the cation selectivity of SAL, which can be shown in the descending trend as K^+^ > Na^+^ > Cs^+^ >> Ca^2+^ [11]. SAL has also demonstrated biological activities in other anti-tumoral actions, such as increasing the death of lung cancer stem cells, sensitising cancer cells to radiation and synergising with dichloroacetate cytotoxicity in colon cancer cells [12,13,14].

Mass spectrometry (MS) plays a key role in the analysis of polyether ionophores [15], particularly in the determination of their possible presence in animal-related bioproducts and human plasma [16,17,18]. When coupled with high-performance liquid chromatography (HPLC), tandem mass spectrometry (MS/MS) has proven to be powerful for the identification of polyether ionophores in many previous studies [19]. Compared to other polyether ionophore antibiotics, fragmentation studies of SAL are lacking. The first MS study of the SAL monosodiated complex and its derivatives was published in 1976 [20]. Here, the characteristic cleavage at ring C of the tricyclic spiroketal system was reported. It was also stated that the sodium cation was firmly chelated with the residual oxygen moieties of the molecule. Another study from 2003 proposed a streamlined schematic representation and fragmentation study of the SAL mono-sodiated and doubly sodiated molecular ions [21]. However, detailed fragmentation studies of SAL using high resolution accurate mass (HRAM) sequential mass spectrometry (MS^n^) and ion mobility spectrometry (IMS) MS have never been published.

Another difficulty in the analysis of SAL is its tendency to isomerise in water–methanol solution at ambient temperature (see Figure 1) [22]. There is currently little MS data on the isomeric form in the literature. Due to its potential development as a human pharmaceutical resulting from the anticancer stem cell functionality, understanding the fragmentation pathways of both the original and isomeric form is crucial. Therefore, in this paper, we proposed a complete structural characterisation and fragmentation pathways for SAL and its isomeric form (iSAL) using HRAM-MS^n^. Product ions generated from the different isomeric forms of SAL were also successfully identified and differentiated using a combination of HRAM-MS^n^ and IMS-MS/MS. Structures were visualised by Gaussian computational modelling, and the results were rationalised by calculation of collision cross-sections (CCSs).

## 2. Results and Discussion

### 2.1. Overview

During the previous MS studies of SAL, three types of fragmentation were reported. These were a characteristic cleavage occurring between C-17 and C-21 across ring C of the tricyclic spiroketal system, cleavage at the hydroxyketone group (C-11) by a McLafferty-type rearrangement [23], and at positions of the cyclic ether rings [20]. Later nomenclature uses type A and F to describe fragmentations observed with SAL where either of the termini remain intact [24]. Product ions with the carboxyl terminal remaining intact are termed type A, whereas ions with the hydroxyl group terminal remaining intact are termed type F. Due to the complexity of the fragmentation pathways observed, the authors here propose the use of additional nomenclature for clarification. These ions are denoted as ‘MC_X,Y_A or F’ or ‘MiC_X,Y_A or F’. M means that the fragmentation was initiated through a McLafferty-type rearrangement, which is predominately seen in the MS^n^ spectra of SAL. The lower case i represents the product ions were from iSAL. C_X,Y_ identifies which carbons (superscript number X and Y), and where the C-C bonds were broken. Finally, the A or F subscript signifies whether the product ion is produced by a type A or type F fragmentation. Based on this nomenclature, the Orbitrap MS^n^ spectra from collision-induced dissociation (CID) of SAL are investigated, and the observed product ions are tabulated (see Tables S1 and S2 in Supplementary Information). The nature of the isomerisation of SAL complicates the spectra, making the structural characterisation more challenging. Table S1 summarises all the product ions identified and distinguished, their precursor ions (SAL or iSAL) and their respective fragmentation pathways. Table S2 lists the occurrence of all product ions throughout the MS^n^ analysis, along with formulae assignments and mass accuracies.

### 2.2. MS^n^ of Salinomycin, m/z 773

**Figure 2:**
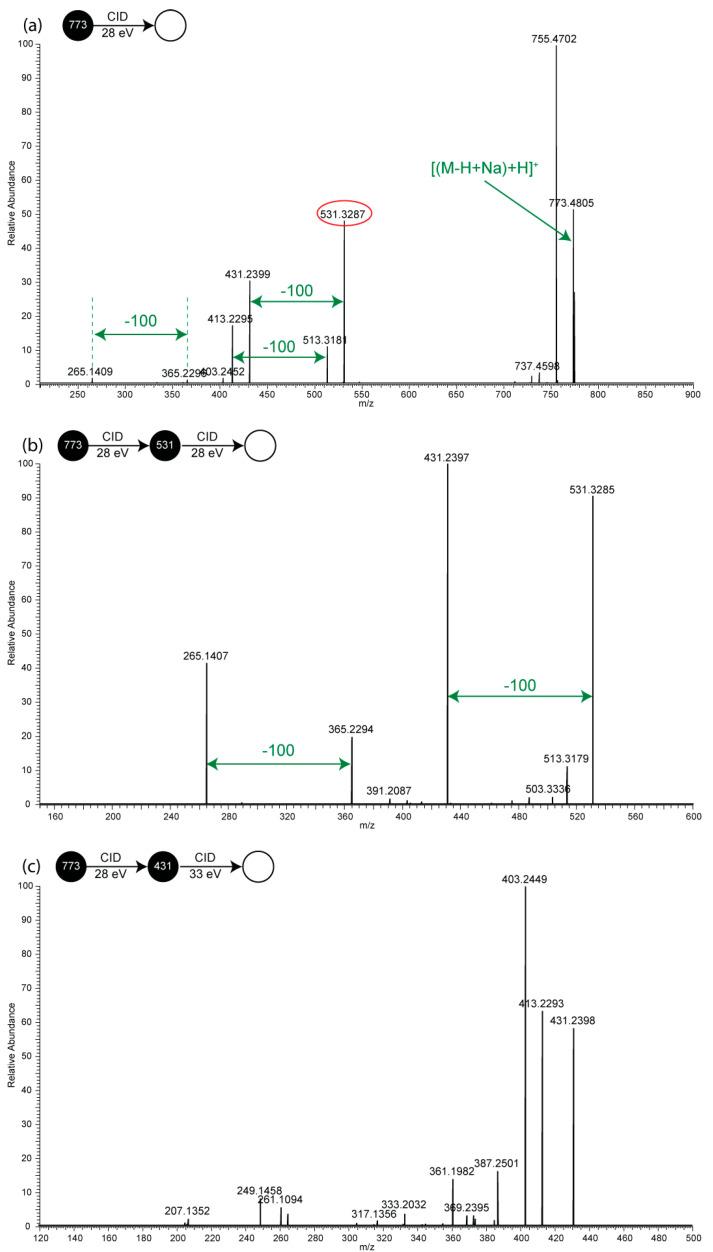
Positive ion ESI-CID-MS^n^ spectra of SAL. (**a**) MS/MS spectrum of [(SAL−H+Na)+H]^+^ (PI *m*/*z* 773); (**b**) MS^3^ spectrum of [(SAL−H+Na)+H]^+^ (PI *m*/*z* 531); (**c**) MS^3^ spectrum of [(SAL−H+Na)+H]^+^ (PI *m*/*z* 431). The routes to the ions and collision energies used are indicated on each spectrum. In spectrum (**a**,**b**) the 100 *m*/*z* losses are also highlighted in green.

Upon initial examination of the MS^n^ spectra of the protonated salinomycin sodium complex [(M−H)+Na+H]^+^ (see Figure 2) precursor ion (PI) *m*/*z* 773, the extensive losses of H_2_O, CO and CO_2_ are readily seen. This is expected based on the structures of SAL and its isomer iSAL. Secondly, type F fragmentation dominates the spectra of SAL and iSAL. Type A fragmentation produced more ions towards the lower mass range (*m*/*z* < 365). Especially with *m*/*z* 265, all of the MS^n^ product ions of *m*/*z* 265 resulted from type A fragmentation. Lastly, the distinct fragmentation pathway of iSAL results in product ions that are all from type F fragmentation. Firstly, the main reason for this is that the structure of iSAL from C-1 to C-13 is identical to that of SAL, and as a result, fragmentation originating from this part of the molecule produces indistinct type A product ions by examination of the HRAM MS^n^ spectra alone. Secondly, this can be explained by the ring opening of rings B, C and D in iSAL, offering a higher degree of freedom leading to entropically favoured fragmentation. These observations are useful in differentiating between the product ions of SAL and iSAL and are, as a result, crucial in proposing a full, reliable fragmentation schemes for both SAL and iSAL.

Figure 2a is the MS/MS spectrum of SAL. Typical product ions to be investigated further are *m*/*z* 531, 431 and 265 (see Figure 2b,c). These product ions are essential in determining which precursor ion isomer they were generated from, and via which type of fragmentation pathway. There are two consecutive losses of water from *m*/*z* 773, and losses of CO_2_ are also present, although the intensities of water losses are much higher. Notice the 100 mass unit differences between peak *m*/*z* 531 to 431, *m*/*z* 513 to 413, and *m*/*z* 365 to 265. These result from a characteristic fragmentation pathway in the MS^n^ of SAL and are essential in differentiating between SAL and iSAL.

### 2.3. Consideration of Product Ion m/z 531

**Scheme 1:**
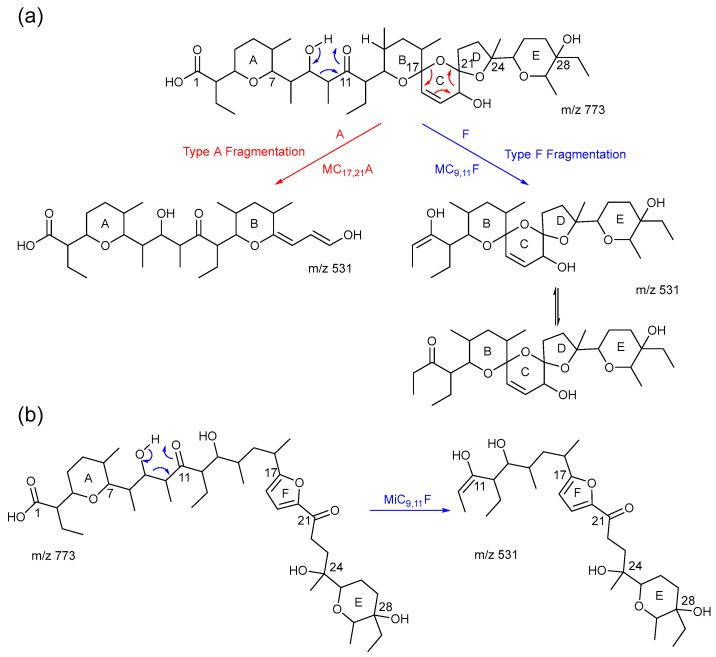
The proposed mechanisms for the generation of the three isomeric product ions at *m*/*z* 531 of (**a**) salinomycin sodiated ion and (**b**) its isomer. Type A ions are marked in red, and type F ions are marked in blue. The sodium cation is left out for clarity.

Three isomeric product ions (observed at *m*/*z* 531—highlighted in red in Figure 2a) are generated directly from *m*/*z* 773 by one of three competing fragmentation pathways (see Scheme 1). On the right-hand side of the scheme is the type F fragmentation, resulting from a McLafferty-type rearrangement between C-9 and C-11. The carbonyl oxygen forms a cyclical structure by hydrogen bonding, which is marked in blue. Alternatively, on the left-hand side, a type A *m*/*z* 531 can be produced by a similar McLafferty-type rearrangement but this time in ring C (marked in red). In the case of the iSAL, *m*/*z* 531 is solely produced by type F fragmentation. The reason for this is the presence of the furan ring (ring F) in the iSAL structure. To obtain *m*/*z* 531 by type A fragmentation, this furan ring would have to be broken, which is extremely energetically unfavourable. The presence of the three isomeric *m*/*z* 531 product ions demonstrates the complexity of the fragmentation, which can be easily overlooked [25].

### 2.4. The Product Ions of m/z 531

**Scheme 2:**
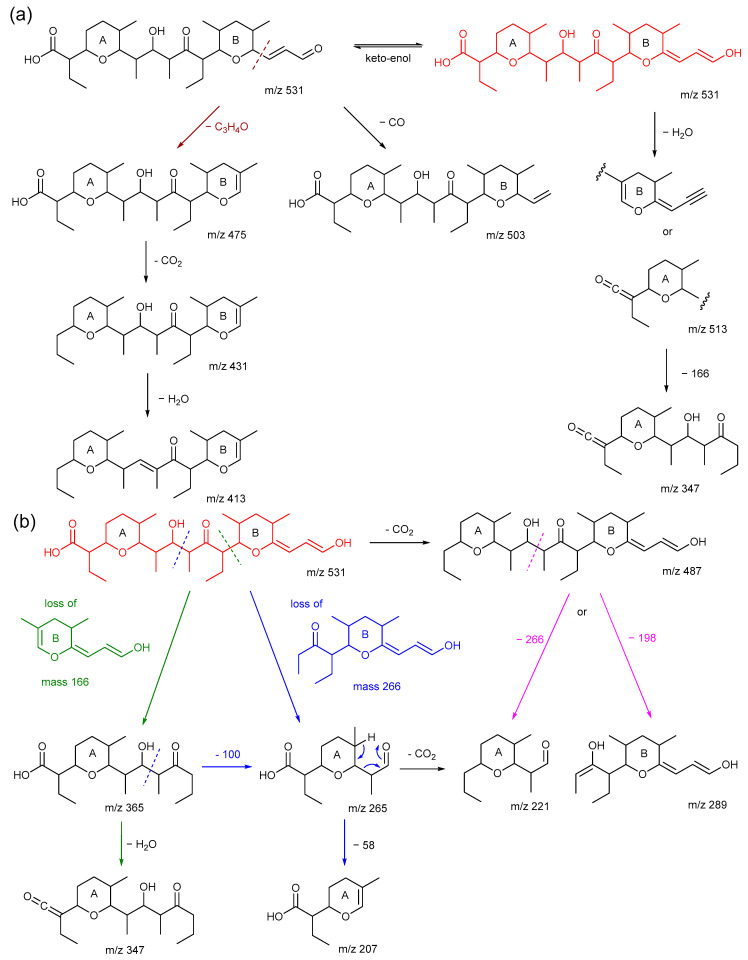
The proposed mechanism for the MS^n^ type A fragmentation of precursor ion *m*/*z* 531. Product ions are *m*/*z* 513, 503, 365, 347, 289, 265, 221, 207 with common neutral losses of water, CO, CO_2_ and mass 100, 166 and 266. Route (**a**) is generated by cleavages adjacent to ring **B** and route (**b**) is generated by cleavages between rings **A** and **B**. The sodium cation is left out for clarity.

**Scheme 3:**
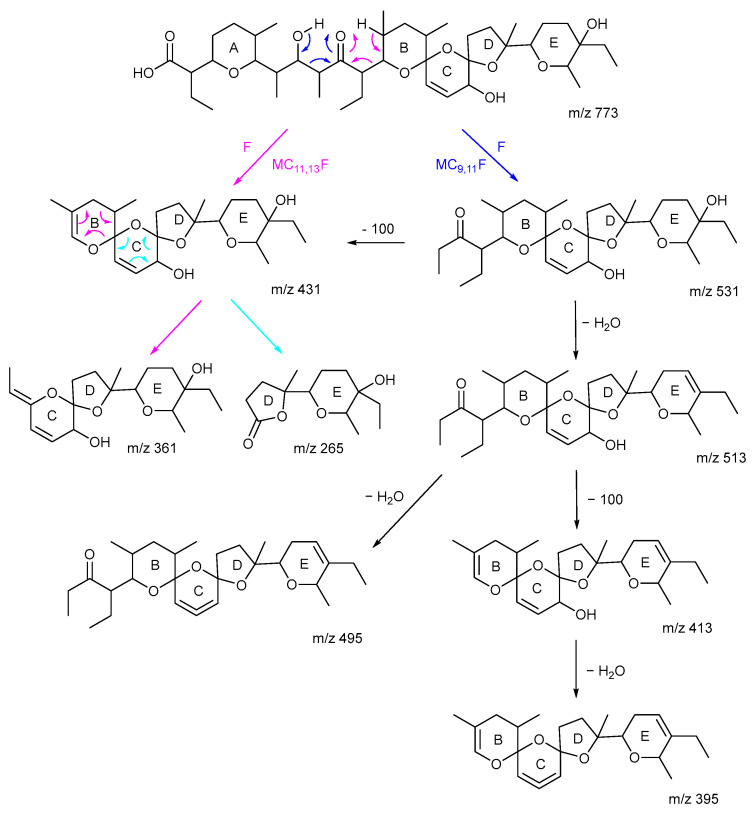
The proposed mechanism for the MS^n^ fragmentation of *m*/*z* 773 and *m*/*z* 531 following the type F fragmentation pathway. Product ions are *m*/*z* 513, 495, 431, 413, 395, 361 and 265, with common neutral loss of water and mass 100. The sodium cation is left out for clarity.

**Scheme 4:**
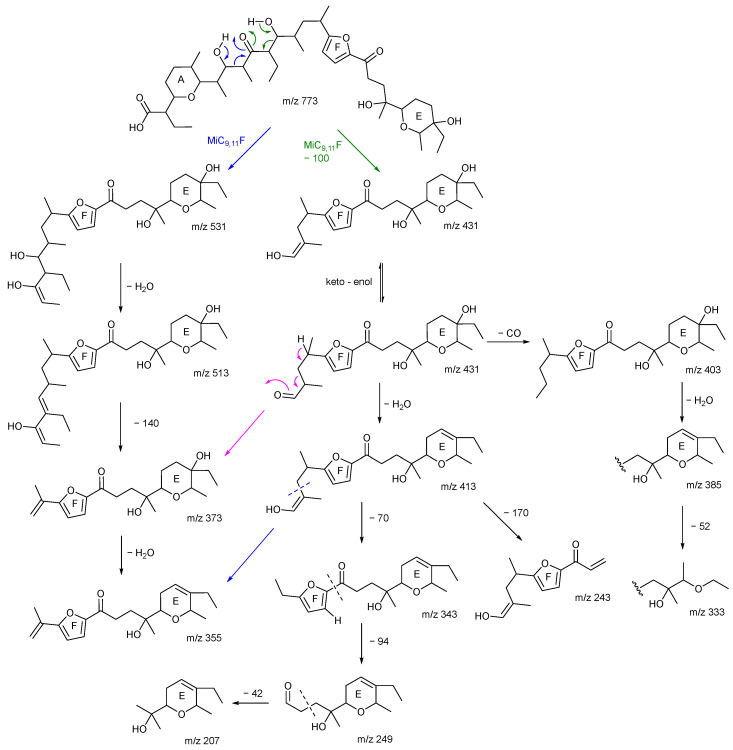
The proposed mechanism for the MS^n^ fragmentation of iSAL in the positive ion mode (MS^2^ precursor ion *m*/*z* 773). Product ions are at *m*/*z* 531, 513, 431, 413, 403, 373, 355, 343, 333, 249, 243 and 207, with common neutral losses of water and CO and various carbon chain cleavages due to hydride shifts. The sodium cation is left out for clarity.

A detailed fragmentation pathway for the MS^n^ fragmentation of *m*/*z* 531 (see Figure 2b for the MS^3^ spectrum) is proposed in Scheme 2, Scheme 3 and Scheme 4. The multiple isomers of *m*/*z* 531 are impossible to separate in direct infusion MS^n^, and initially it may seem that many of the product ions can be formed from any of the three forms. Product ions that are unique to iSAL are colour-coded in Table S1. The proposed fragmentation pathways are included in Scheme 2, Scheme 3 and Scheme 4 based on their types of fragmentations. Scheme 2 shows the product ions that are produced via the type A fragmentation from *m*/*z* 531.

The first CO_2_ loss to produce product ion *m*/*z* 487 was expected due to the presence of the carboxyl terminus in the type A isomer of *m*/*z* 531. This ion can subfragment to produce either *m*/*z* 289 or 221, depending on which end chelates the sodium (pink routes). Product ion *m*/*z* 513 could result from H_2_O elimination from either the carboxyl or secondary alcoholic hydroxyl ends, but only the carboxyl elimination can produce *m*/*z* 347. Product ion *m*/*z* 365 results from a direct cleavage between C-12 to C-13 (green route), leaving ring B propan-2-en-1-ol as a neutral loss. The most interesting observation is product ion *m*/*z* 265 (blue route) which carries on producing product ions at *m*/*z* 221 and *m*/*z* 207. The subfragmentations observed for product ion *m*/*z* 265 is a key tool in identifying which route it was generated from, and in determining the structure of its precursor ion *m*/*z* 531. As *m*/*z* 265 could also be produced via type F fragmentation but it would not be able to produce further subfragmentations due to its different structure (see Scheme 3); this is discussed in the next section. This observation, combined with further data, provides evidence that MS^n^ is a powerful tool in differentiating structural isomers.

Scheme 3 shows the proposed fragmentation mechanism for the MS^n^ analysis of *m*/*z* 531 following the type F pathway. The routes marked in blue and black indicate that the product ions have the hydroxyl terminus intact. The initial cleavage results in *m*/*z* 531 and *m*/*z* 431 (see nomenclature for clarity). Product ion *m*/*z* 531 undergoes a McLafferty-type rearrangement resulting in the breakage of C-C bonds and then *m*/*z* 431 via a pericyclic fragmentation. Product ion *m*/*z* 431 could also result from *m*/*z* 531 via the loss of the ketone (mass 100). Multiple H_2_O losses can occur from *m*/*z* 531 and its product ions, and these are listed in Table S1 and not included in Scheme 3 for clarity. Product ions *m*/*z* 361 and *m*/*z* 265 can also result from *m*/*z* 531. These are differentiated by the colour of their proposed mechanisms. However, this product ion *m*/*z* 265 isomer does not undergo any further losses, unlike the type A product ion *m*/*z* 265 isomer (see Scheme 2). The cyclical structure of *m*/*z* 265 on the type F route likely prevents it from fragmentating further due to the stabilisation provided by the chelated sodium cation.

Scheme 4 is the proposed mechanism for the MS^n^ fragmentation of iSAL following the type F pathway (see Table S1). This fragmentation pathway is in competition with the type A and F pathways of SAL. Other than the iconic *m*/*z* 531 and *m*/*z* 431 product ions, iSAL generates unique product ions through its open ring backbone such as *m*/*z* 403, 395, 243, etc. These ions are only present in the MS^n^ of iSAL. They are useful in determining the fragmentation pathway of the isomeric form of salinomycin. Due to the presence of multiple secondary and tertiary alcohols, loss of water is common, and the position of each water loss defines which route the fragmentation takes next.

### 2.5. IMS-MS/MS of Salinomycin

Ion mobility spectroscopy (IMS)-based studies on natural products have been increasing in popularity in recent research. In combination with high-resolution MS^n^, IMS provides a powerful tool for structure analysis and qualitative investigation of natural products [25,26,27]. Separation was performed based on differences in drift times for SAL and iSAL. And based on these results, we have for the first time proposed new structural forms of SAL with variations in the location of the sodium binding. These are named ‘ionoforms’ using a similar etymology as for the established word ‘protomers’, which is used to describe protonated ions with different proton positions [28]. The word ‘ionoform’ refers to ‘different forms of an ion’ and is here defined as isomers of metal cation complexes with variations solely in the chelation position of the cation. Previous studies show that ionophores such as Monensin produced di-sodiated complexes in agreement with our observation of di-sodiated salinomycin [29]. It was hypothesised that the high metal binding affinity of SAL would permit the metal cation to chelate in various positions of the molecule. For example, the Na^+^ could be chelated towards either end of the molecule. In this way, the differences in the chelation position would explain the fundamental differences in the fragmentation routes being followed, i.e., type A or type F routes. Therefore, it is important to be able to distinguish between these ionoforms. Herein, we present IMS-MS/MS data with computational modelling to rationalise these observations.

**Figure 3:**
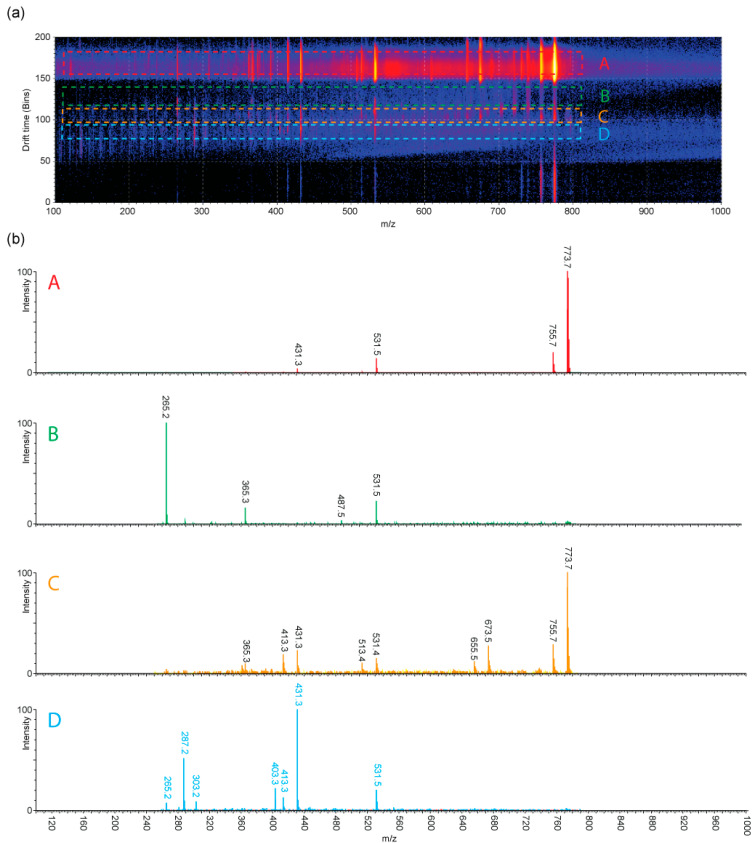
Positive ion mode ESI-IMS-MS/MS data for [(SAL−H+Na)+H]+ at transfer energy 40 eV. Panel (**a**) shows the drift scope plots of mass vs. drift time, and panel (**b**) shows the mobility-separated MS/MS spectra of *m*/*z* 773. The coloured boxes in (**a**) show the regions of the IMS spectrum used to generate the IMS resolved MS/MS spectra in (**b**).

IMS-MS/MS data was captured for ion transfer energies ranging from 30 eV to 55 eV. This is when MS/MS is performed post-IMS to produce isomeric differentiation on the precursor ions. Figure 3a shows the drift time against the mass for the IMS spectrum of *m*/*z* 773 (SAL precursor ion at 40 eV collision energy in the transfer zone). The interesting observation is the number of [(SAL−H+Na)+H]^+^ peaks and the separation in drift times for the product ion peaks *m*/*z* 531, 431 and 265. The resolved IMS spectra are due to the isomerisation of SAL in solution and its resulting ionoforms in the gas phase. Different rows on the drift scope data are labelled and marked with letters and cross-referenced to their converted IMS separated product ion mass spectra (see Figure 3b).

The number of separations of *m*/*z* 773 provides further insight into our previous observations in the MS^n^ analysis. Multiple fragmentation routes can only be rationalised if SAL has multiple isomers AND ionoforms in the gas phase depending on the location of the sodium cation. Based on previous MS^n^ data and the proposed mechanism of [(SAL−H+Na)+H]^+^, two *m*/*z* 531 product ions are generated from the original [(SAL−H+Na)+H]^+^ via type A and F fragmentations. Then iSAL can also generate a type F ion at *m*/*z* 531. However, due to the structure of iSAL, a type A *m*/*z* 531 was not observed in its MS^n^ spectra. In addition, Figure 3b also differentiates the iSAL spectrum from SAL by the presence of the product ion peak at *m*/*z* 403, which is unique to this isomer. Therefore, the spectra are as follows: spectrum D is iSAL which is demonstrated by its unique product ion at *m*/*z* 403. Spectrum C is [(SAL−H+Na)+H]^+^ type F with the sodium likely chelated at the hydroxy ring end. Spectrum B is [(SAL−H+Na)+H]^+^ type A with the sodium most likely to be chelated at the ring end but could be closer to the carboxyl group at the A terminus. Finally, spectrum A, which belongs to the original [(SAL−H+Na)+H]^+^ where the sodium is chelated in the middle, results in a more stabilised form with the least number of product ions being generated.

**Figure 4:**
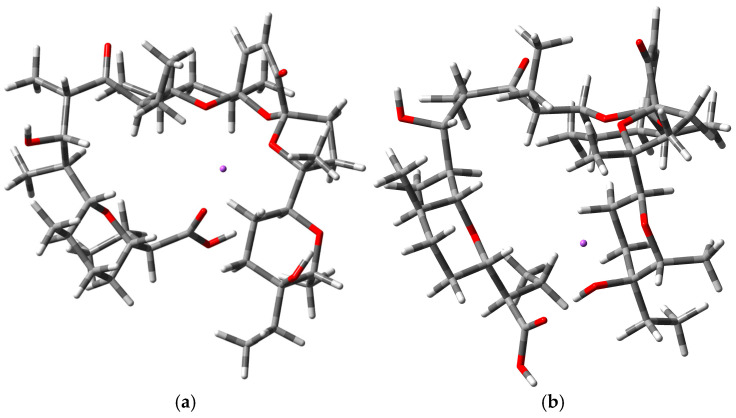
Gaussian modelling of optimised [(SAL−H+Na)+H]^+^ 3D structures. Structure (**a**) shows chelation by the ether oxygens of ring **B**, **C**, and **D** and structure (**b**) shows chelation at the pyran oxygen and **F** terminus. Grey: carbon; white: hydrogen; red: oxygen; and purple: sodium.

Theoretical CCS values were calculated on computational models of [(SAL−H+Na)+H]^+^ and [(iSAL−H+Na)+H]^+^ to aid a more detailed interpretation of the IMS data, as well as the three types of product ion *m*/*z* 531, type A and F from original SAL, and type F from iSAL. [(SAL−H+Na)+H]^+^ ionoforms were modelled as sodium in the middle and sodium at the ring end. The optimised structures of the two ionoforms of [(SAL−H+Na)+H]^+^ are shown in Figure 4.

From the optimised structure (a), the sodium ion is chelated by the ether oxygens on rings B, C, and D, and it also interacts with the carboxyl oxygen at the A terminus. In structure (b), the sodium ion is at the ring end and is chelated by the pyran oxygen and the OH group at the F terminus.

Table 1 shows the calculated CCS values for salinomycin, and this data is purely theoretical and solely used for comparison with experimental observation. Combining these results with Figure 3a established a form of agreement. Ring end sodium [(SAL−H+Na)+H]^+^ has a lower CCS and, therefore, is expected to have a lower drift time. The separation in the drift time figure does not feature the [(iSAL−H+Na)+H]^+^ as a precursor ion. However, the similarities between the CCS values for SAL and iSAL monosodium ion could mean no experimental separation. This supports the IMS-MS/MS data for no observation of the [(iSAL−H+Na)+H]^+^ molecular ion.

**Figure 5:**
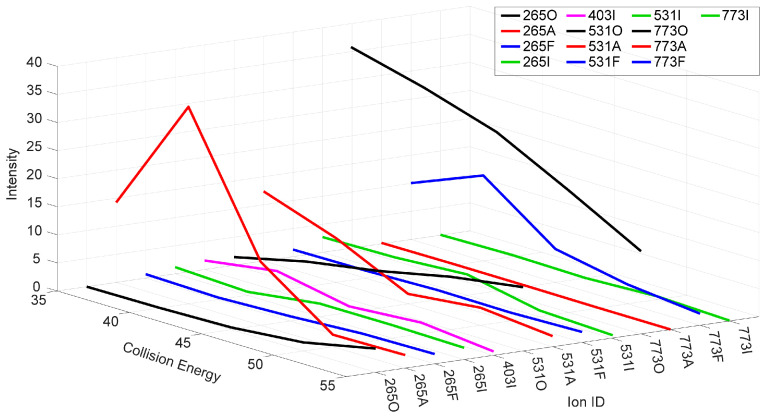
Three-dimensional energy breakdown graph (EBG) for the product ions of SAL obtained from the IMS-MS/MS spectra. The peaks are coloured to aid visibility. O: original SAL (black); A: A-type fragmentation (red); F: F-type fragmentation (blue) and I: isomeric SAL (green and fuchsia).

Energy breakdown graphs (EBG) have been widely applied in MS/MS studies [30,31,32,33], but this is the first time they have been used in a 3D illustration for polyether ionophores such as SAL. EBGs demonstrate the relationship between peak intensity of selected ions and the collision energy. In Figure 5, the relative peak intensities of selected product ions *m*/*z* 531, 403 and 265 are compared alongside the precursor ion *m*/*z* 773 from the different ionoforms. It can be observed that the majority of product ions lose intensity when the collision energy is higher than 40 eV. Secondly, when the peak intensity of the precursor ion *m*/*z* 773 decreases, the peak intensity of their corresponding product ions increases. Lastly, the peak intensity of the product ions resulting from type A fragmentation is considerably higher when compared with those from other routes.

## 3. Materials and Methods

Salinomycin monosodium salt, from *Streptomyces albus*, ≥ 98% was purchased from Sigma-Aldrich (Gillingham, UK) and dissolved in methanol–water (50%) to make up a solution of 10 mg/mL. This solution was diluted to the appropriate concentration immediately prior to analysis. All solvents used throughout the study were gradient grade (Fisher Scientific, Loughborough, UK).

Direct infusion (at 0.1 mg/mL) positive ion mode ESI-MS and ESI-MS^n^ were performed on an Orbitrap Elite mass spectrometer using a HESI source (Thermo Fisher Scientific, Hemel-Hempstead, UK). ESI was performed at a source voltage of 3 kV, source temperature of 350 °C and a capillary temperature of 275 °C. Analyte solutions were introduced into the instrument via a syringe pump at a flow rate of 5 mL/minute. The mass range was set to 100–1000 *m*/*z*, scan rate was 2 scan/s, and the resolution was set to 240,000. CID-MS^n^ using dry N_2_ as the collision gas was performed on the isolated precursor ions (2 *m*/*z* isolation) and the collision energy used is specified in the results. Spectra were achieved by manually summing the scans across the acquisition time range used—usually between 30 s and 1 min. Tuning and calibration were performed using the built-in tuning and calibration routines and Pierce LTQ ESI Calibration Solution (Life Technologies, Paisley, UK).

Direct infusion (1 mg/mL) positive ion mode nanospray MS/MS and IMS-MS/MS were performed on a Synapt G2-Si mass spectrometer (Waters, Manchester, UK) equipped with a Nanomate Triversa chip-based nanospray system (Advion Biosciences, Norwich, UK). The Nanomate was set to aspirate 5 μL of sample solution, which was enough to generate approximately 30 min of usable signal. IMS-MS/MS of the relevant complex was achieved by isolating the precursor, subjecting it to IMS separation and then fragmenting it post IMS using CID-MS/MS in the transfer cell of the travelling-wave ion mobility spectrometer. Collision energies were set from 30 to 50 eV, and spectra were collected individually along with drift time for approximately a minute per sample per collision energy used. All IMS-MS data were analysed using DriftScope (v2.9) and exported to MassLynx (v4.1) to generate spectra.

Gaussian 16 was used to perform all optimisations [34]. Density functional theory (DFT) was used with exchange hybrid functional MPW1PW9. The basis set used for salinomycin sodiated ions and any respective product ions was 6-311G(d,p). Theoretical CCS data of selective salinomycin structures and product ions were calculated by IMoS v.1.10—https://www.imospedia.com/imos, accessed on 22 April 2023 [35].

## 4. Conclusions

In this study, high-resolution, accurate-mass tandem mass spectra were obtained for the salinomycin sodium complex ion. From the MS^n^ data acquired, it was possible to identify and differentiate product ion peaks generated from the original form of salinomycin and its structural isomer. All product ions observed have their formulae assigned by accurate mass (see Table S2), and detailed fragmentation mechanisms are proposed for both isomeric forms of salinomycin. These mechanisms are used to rationalise product ions observed and confirm the proposed fragmentation pathways.

IMS was implemented to achieve the successful structural separation of salinomycin and its various forms for the first time. From this, Gaussian was used to model sodiated salinomycin, its ionoforms and the isomer. It was also used to optimise structures of product ions generated from type A and type F fragmentation. These modelling results were then adopted in calculating theoretical collision cross-sections for selected ions. The results match the experimentally determined drift times seen in the IMS spectra, confirming our theory that salinomycin exists in more than one structural form (ionoforms) when chelated with sodium, which is crucial in understanding that the position of the sodium chelation has fundamental effects on the fragmentation routes being followed and product ions being generated. Hence, the observation of the multiple ionoforms of salinomycin enables the rationalisation of both type A and type F product ions in the MS^n^ spectra. This would be otherwise unachievable with conventional MS methods. The combination of IMS-MS/MS and HRAM-MS^n^ is vital in the recognition of the complexity of MS^n^ analysis of natural products and drug target molecules. Selected product ions were combined into a 3D energy breakdown graph to track the changes in peak intensity vs. collision energy. The results match our prediction and further consolidate the effectiveness of using energy breakdown graphs in MS/MS studies when a series of collision energies were used [33].

This study demonstrates, for the first time, the combined efficacy of a mixture of analytical techniques in the analysis of complex molecules such as polyether ionophore salinomycin. By combining high-resolution mass spectrometry methods such as MS^n^ and IMS with computational modelling, structural isomers of salinomycin could be characterised and their fragmentation pathways rationalised. The results provide invaluable insight into the structural behaviour of salinomycin in solution, its chelation to sodium and fragmentation behaviour in the gas phase. We believe that these results will be extremely useful in broadening pharmaceutical studies of salinomycin and its anticancer properties. The methodologies adopted in this study could also be applied to the thorough analysis of a variety of polyether ionophores and their metabolites.

## Figures and Tables

**Table 1:** Calculated CCS values for [(SAL−H+Na)+H]+, its isomer and ionoform, and three *m*/*z* 531 product ion types.

Name	Position of Na Cation	Calculated CCS (A^2^)
[(SAL−H+Na)+H]^+^	Middle	283.58
[(SAL−H+Na)+H]^+^	Ring End	277.28
[(iSAL-H+Na]+H]^+^	Middle	285.54
531 Na iF	-	261.25
531 Na A	-	241.59
531 Na F	-	225.07

## Data Availability

The raw data is available from the University of Bristol data repository, data.bris, at https://doi.org/10.5523/bris.34i1k2ds4u8zh2ecrhp0q4mmdp.

## Supplementary Materials

The following supporting information can be downloaded at https://www.mdpi.com/article/10.3390/molecules30234512/s1.

## Abbreviations

The following abbreviations are used in this manuscript:

CID	Collision Induced Dissociation
CCS	Collision Cross-Section
DFT	Density Functional Theory
EBG	Energy Breakdown Graph
ESI	Electrospray Ionisation
HESI	Heated Electrospray Ionisation
HPLC	High-Performance Liquid Chromatography
HRAM	High Resolution Accurate Mass
IMS	Ion Mobility Spectrometry
iSAL	Isomeric Salinomycin
M	Neutral Salinomycin Molecule
MS	Mass Spectrometry
MS/MS	Tandem Mass Spectrometry
MS^n^	Sequential Mass Spectrometry
SAL	Salinomycin

## References

[1] Miyazaki Y., Shibata A., Yahagi T., Hara M., Hara K., Yoneda S., Kasahara H., Nakamura Y. (1980). Method of Producing Salinomycin Antibiotics. U.S. Patent.

[2] Anadón A., Martínez-Larrañaga M.R., Motarjemi Y. (2014). Veterinary drugs residues: Coccidiostats. Encyclopedia of Food Safety.

[3] Martins R.R., Silva L.F.G., Pereira A.M.P.T., Esteves A., Duarte S.C., Pena A. (2022). Coccidiostats and poultry: A comprehensive review and current legislation. Foods.

[4] Sudhakara Reddy B., Sivajothi S., Rayulu V.C. (2015). Clinical coccidiosis in adult cattle. J. Parasit. Dis..

[5] Jolley W.R., Bardsley K.D. (2006). Ruminant coccidiosis. Vet. Clin. Food Anim. Pract..

[6] Cockrell R.S., Harris E.J., Pressman B.C. (1966). Energetics of potassium transport in mitochondria induced by valinomycin. Biochemistry.

[7] Gupta P.B., Onder T.T., Jiang G., Tao K., Kuperwasser C., Weinberg R.A., Lander E.S. (2009). Identification of selective inhibitors of cancer stem cells by high-throughput screening. Cell.

[8] Wang H., Zhang H., Zhu Y., Wu Z., Cui C., Cai F. (2021). Anticancer mechanisms of salinomycin in breast cancer and its clinical applications. Front. Oncol..

[9] Koltai T., Reshkin S.J., Harguindey S. (2020). An Innovative Approach to Understanding and Treating Cancer: Targeting pH.

[10] Naujokat C., Steinhart R. (2012). Salinomycin as a drug for targeting human cancer stem cells. J. Biomed. Biotechnol..

[11] Pressman B.C. (1976). Biological applications of ionophores. Annu. Rev. Biochem..

[12] Larzabal L., El-Nikhely N., Redrado M., Seeger W., Savai R., Calvo A. (2013). Differential effects of drugs targeting cancer stem cell (CSC) and non-CSC populations on lung primary tumors and metastasis. PLoS ONE.

[13] Zhou S., Wang F., Wong E.T., Fonkem E., Hsieh T.C., Wu J.M., Wu E. (2013). Salinomycin: A novel anti-cancer agent with known anti-coccidial activities. Curr. Med. Chem..

[14] Skeberdytė A., Sarapinienė I., Aleksander-Krasko J., Stankevičius V., Sužiedėlis K., Jarmalaitė S. (2018). Dichloroacetate and salinomycin exert a synergistic cytotoxic effect in colorectal cancer cell lines. Sci. Rep..

[15] Rokka M., Jestoi M., Peltonen K. (2013). Trace level determination of polyether ionophores in feed. BioMed Res. Int..

[16] Li Y., Fang J., Wu S., Ma K., Li H., Yan X., Dong F. (2010). Identification and quantification of salinomycin in intoxicated human plasma by liquid chromatography–electrospray tandem mass spectrometry. Anal. Bioanal. Chem..

[17] Blanchflower W.J., Kennedy D.G. (1996). Determination of monensin, salinomycin and narasin in muscle, liver and eggs from domestic fowl using liquid chromatography-electrospray mass spectrometry. J. Chromatogr. B Biomed. Sci. Appl..

[18] Mortier L., Daeseleire E., Peteghem C.V. (2005). Determination of the ionophoric coccidiostats narasin, monensin, lasalocid and salinomycin in eggs by liquid chromatography/tandem mass spectrometry. Rapid Commun. Mass Spectrom..

[19] Spisso B.F., Ferreira R.G., Pereira M.U., Monteiro M.A., Cruz T.A., Costa R.P., Lima A.M.B., Nóbrega A.W. (2010). Simultaneous determination of polyether ionophores, macrolides and lincosamides in hen eggs by liquid chromatography–electrospray ionization tandem mass spectrometry using a simple solvent extraction. Anal. Chim. Acta.

[20] Kinashi H., Ōtake N. (1976). An interpretation of the mass spectra of salinomycin and its derivatives. Agric. Biol. Chem..

[21] Miao X.-S., March R.E., Metcalfe C.D. (2003). Fragmentation study of salinomycin and monensin A antibiotics using electrospray quadrupole time-of-flight mass spectrometry. Rapid Commun. Mass Spectrom..

[22] Davis A.L., Harris J.A., Russell C.A., Wilkins J.P.G. (1999). Investigations by HPLC-electrospray mass spectrometry and NMR spectroscopy into the isomerisation of salinomycin. Analyst.

[23] McLafferty F.W. (1959). Mass spectrometric analysis. Molecular rearrangements. Anal. Chem..

[24] Kim Y.H., Yoo J.S., Lee C.H., Goo Y.M., Kim M.S. (1996). Application of fast atom bombardment combined with tandem mass spectrometry to the structural elucidation of O-demethylabierixin and related polyether antibiotics. J. Mass Spectrom..

[25] Masike K., Stander M.A., de Villiers A. (2021). Recent applications of ion mobility spectrometry in natural product research. J. Pharm. Biomed. Anal..

[26] Carnevale Neto F., Clark T.N., Lopes N.P., Linington R.G. (2022). Evaluation of ion mobility spectrometry for improving constitutional assignment in natural product mixtures. J. Nat. Prod..

[27] Stow S.M., Lareau N.M., Hines K.M., McNees C.R., Goodwin C.R., Bachmann B.O., Mclean J.A., Havlíček V., Spížek J. (2014). Natural Products Analysis: Instrumentation, Methods, and Applications.

[28] Warnke S., Seo J., Boschmans J., Sobott F., Scrivens J.H., Bleiholder C., Bowers M.T., Gewinner S., Schöllkopf W., Pagel K. (2015). Protomers of benzocaine: Solvent and permittivity dependence. J. Am. Chem. Soc..

[29] Lopes N.P., Stark C.B.W., Hong H., Gates P.J., Staunton J. (2002). Fragmentation studies on monensin A and B by accurate-mass electrospray tandem mass spectrometry. Rapid Commun. Mass Spectrom..

[30] Butcher C.P.G., Dyson P.J., Johnson B.F.G., Langridge-Smith P.R.R., McIndoe J.S., Whyte C. (2002). On the use of breakdown graphs combined with energy-dependent mass spectrometry to provide a complete picture of fragmentation processes. Rapid Commun. Mass Spectrom..

[31] Mörlein S., Schuster C., Paal M., Vogeser M. (2020). Collision energy-breakdown curves—An additional tool to characterize MS/MS methods. Clin. Mass Spectrom..

[32] Murakami T., Iwamuro Y., Ishimaru R., Chinaka S., Kato N., Sakamoto Y., Sugimura N., Hasegawa H. (2019). Energy-resolved mass spectrometry for differentiation of the fluorine substitution position on the phenyl ring of fluoromethcathinones. J. Mass Spectrom..

[33] Jiang C., Gates P.J. (2024). Systematic characterisation of the fragmentation of flavonoids using high-resolution accurate mass electrospray tandem mass spectrometry. Molecules.

[34] Frisch M.J., Trucks G.W., Schlegel H.B., Scuseria G.E., Robb M.A., Cheeseman J.R., Scalmani G., Barone V., Petersson G.A., Nakatsuji H. (2016). Gaussian 16, Revision B.01.

[35] Larriba-Andaluz C., Hogan C.J. (2014). Collision cross section calculations for polyatomic ions considering rotating diatomic/linear gas molecules. J. Chem. Phys..

