# Supplementary material for: High-Resolution Electrospray and Ion Mobility Sequential Mass Spectrometry for Structural Characterisation of Anticancer Stem Cell Agent Salinomycin and Its Isomers
Source: Molecules. 2025 Nov 22;30(23):4512. doi: 10.3390/molecules30234512 (PMC12693351; doi:10.3390/molecules30234512)
Supplement: Supplementary file 1 [file molecules-30-04512-s001.zip › molecules-3934273-supplementary.pdf]

High-Resolution Electrospray and Ion Mobility Sequential Mass Spectrometry for Structural Characterisation of Anticancer Stem Cell Agent Salinomycin and its Isomers.

Candy Jiang and Paul J. Gates*

School of Chemistry, University of Bristol, Cantock's Close, Bristol, BS8 1TS, United Kingdom (* paul.gates@bristol.ac.uk).

Supplementary Materials

Contents

Table S1. Table of $[(SAL\text{-}H+Na)+H]^+$ and $[(iSAL\text{-}H+Na)+H]^+$ as the precursor ions and corresponding identified product ions, their fragmentation pathways and ion identities from MS^n spectra in the positive ion mode. Fragmentation pathway is colour coded based on the routes. Red: Type A fragmentation leaving carboxyl end; Blue: Type F fragmentation leaving hydroxyl end and Green: Fragmentation from isomeric SAL (iSAL).

Table S2. Table of observed *m/z*, formulae, theoretical *m/z*, error (ppm) and ion identification from the MS^n analysis of SAL $[(SAL\text{-}H+Na)+H]^+$.

Figure S1. Positive ion ESI-CID-MS^3 spectrum of $[(SAL\text{-}H+Na)+H]^+$ (PI *m/z* 755).

Figure S2. Positive ion ESI-CID-MS^3 spectrum of $[(SAL\text{-}H+Na)+H]^+$ (PI *m/z* 513).

Figure S3. Positive ion ESI-CID-MS^4 spectrum of $[(SAL\text{-}H+Na)+H]^+$ (PI *m/z* 431).

Figure S4. Positive ion ESI-CID-MS^3 spectrum of $[(SAL\text{-}H+Na)+H]^+$ (PI *m/z* 413).

Figure S5. Positive ion ESI-CID-MS^4 spectrum of $[(SAL\text{-}H+Na)+H]^+$ (PI *m/z* 413).

Figure S6. Positive ion ESI-CID-MS^4 spectrum of $[(SAL\text{-}H+Na)+H]^+$ (PI *m/z* 365).

Figure S7. Positive ion ESI-CID-MS^4 spectrum of $[(SAL\text{-}H+Na)+H]^+$ (PI *m/z* 265).

Table S1. Table of $[(SAL\text{-}H+Na)+H]^+$ and $[(iSAL\text{-}H+Na)+H]^+$ as the precursor ions and corresponding identified product ions, their fragmentation pathways and ion identities from MS^n spectra in the positive ion mode. Fragmentation pathway is colour coded based on the routes. Red: Type A fragmentation leaving carboxyl end; Blue: Type F fragmentation leaving hydroxyl end and Green: Fragmentation from isomeric SAL (iSAL).

Fomula	m/z	Ion Identity	Fragmentation Type	Pathway
$C_{42}H_{70}O_{11}Na^+$	773	$[(SAL\text{-}H+Na)+H]^+$	Monosodiated Ion	FULL SCAN
	773	$[(iSAL\text{-}H+Na)+H]^+$	Monosodiated Ion	FULL SCAN
$C_{42}H_{68}O_{10}Na^+$	755	$[773\text{-}H_2O]^+$/SAL and iSAL	A and F	MS/MS 773
$C_{42}H_{66}O_9Na^+$	737	$[773\text{-}2H_2O]^+$/SAL and iSAL	A and F	MS/MS 773
$C_{41}H_{70}O_9Na^+$	729	$[773\text{-}CO_2]^+$	**F**	MS/MS 773
$C_{41}H_{68}O_8Na^+$	711	$[755\text{-}CO_2]^+$	**F**	MS/MS 773
$C_{29}H_{48}O_7Na^+$	531	$[773\text{-}242]^+$	${}_{M}C^{17,21}{}_{A}$	MS/MS 773
	531	$[773\text{-}242]^+$	${}_{M}C^{9,11}{}_{F}$	MS/MS 773
	i531	$[i773\text{-}242]^+$	${}_{Mi}C^{9,11}{}_{F}$	MS/MS 773
$C_{29}H_{46}O_6Na^+$	513	$[531\text{-}H_2O]^+$	${}_{M}C^{9,11}{}_{F}$ - 18	MS/MS 773
	i513	$[i531\text{-}H_2O]^+$	${}_{Mi}C^{9,11}{}_{F}$ - 18	MS/MS 773
$C_{29}H_{44}O_5Na^+$	495	$[531\text{-}2H_2O]^+$	${}_{M}C^{9,11}{}_{F}$ - 36	MS^3 773-755
	i495	$[i531\text{-}2H_2O]^+$	${}_{Mi}C^{9,11}{}_{F}$ - 36	MS^3 773-755
$C_{28}H_{48}O_6Na^+$	503	$[531\text{-}CO]^+$	${}_{M}C^{17,21}{}_{A}$ - 28	MS^3 773-531
$C_{28}H_{48}O_5Na^+$	487	$[531\text{-}CO_2]^+$	${}_{M}C^{17,21}{}_{A}$ - 44	MS^3 773-531
$C_{23}H_{36}O_6Na^+$	431	$[531\text{-}100]^+$	${}_{M}C^{9,11}{}_{F}$ - 100	MS/MS 773
	431	[773-342]	${}_{M}C^{11,13}{}_{F}$	MS/MS 773
	i431	$[i531\text{-}100]^+$	${}_{Mi}C^{9,11}{}_{F}$ - 100	MS/MS 773
$C_{23}H_{34}O_5Na^+$	413	$[431\ \text{-}H_2O]^+$	${}_{M}C^{9,11}{}_{F}$ - 100 - 18	MS/MS 773
	i413	$[i431\ \text{-}H_2O]^+$	${}_{Mi}C^{9,11}{}_{F}$ - 100 -18	MS/MS 773
$C_{22}H_{36}O_5Na^+$	i403	$[i431\text{ - }CO]^+$	${}_{Mi}C^{9,11}{}_{F}$ - 100 -28	MS^4 773-531-431
$C_{23}H_{32}O_4Na^+$	395	$[413\text{-}H_2O]^+$	${}_{M}C^{9,11}{}_{F}$ - 100 - 36	MS^3 773-413
	i395	$[i413\text{-}H_2O]^+$	${}_{Mi}C^{9,11}{}_{F}$ - 100 -36	MS^3 773-413
$C_{20}H_{30}O_5Na^+$	i373	$[i513\text{-}140]^+$	${}_{Mi}C^{9,11}{}_{F}$ - 18 - 140	MS^4 773-531-513
$C_{19}H_{34}O_5Na^+$	365	$[531\text{-}166]^+$	${}_{M}C^{17,21}{}_{A}$ - 166	MS^3 773-531
$C_{19}H_{30}O_5Na^+$	361	$[431\text{-}70]^+$	${}_{M}C^{9,11}{}_{F}$ - 100-70	MS^4 773-531-431
$C_{19}H_{32}O_4Na^+$	347	$[513\text{-}166]^+$	${}_{M}C^{9,11}{}_{A}$ - 18 - 166	MS^4 773-531-513
$C_{19}H_{28}O_4Na^+$	i343	$[i413\text{-}70]^+$	${}_{Mi}C^{9,11}{}_{F}$ - 100 -18-70	MS^4 773-755-513
$C_{17}H_{26}O_5Na^+$	i333	$[i403\text{-}70]^+$	${}_{Mi}C^{9,11}{}_{F}$ - 100 -28-70	MS^4 773-531-431
$C_{16}H_{26}O_3Na^+$	289	$[487\text{-}198]^+$	${}_{M}C^{17,21}{}_{A}$ - 44 - 198	MS^3 773-531
$C_{13}H_{22}O_4Na^+$	265	$[531\text{-}266]^+$	${}_{M}C^{17,21}{}_{A}$ - 266	MS^3 773-531
	265	$[431\text{-}166]^+$	${}_{M}C^{9,11}{}_{F}$ - 100 - 166	MS^3 773-431
$C_{13}H_{16}O_3Na^+$	i243	$[i343\text{-}100]^+$	${}_{Mi}C^{9,11}{}_{F}$ - 100 -18-70-100	MS^4 773-755-413
$C_{12}H_{22}O_2Na^+$	221	$[265\text{-}CO_2]^+$	${}_{M}C^{17,21}{}_{A}$ - 266 - 44	MS^4 773-531-265
$C_{10}H_{16}O_3Na^+$	207	$[265\text{-}58]^+$	${}_{M}C^{17,21}{}_{A}$ - 266 - 58	MS^4 773-531-265

Table S2. Table of observed *m/z*, formulae, theoretical *m/z*, error (ppm) and ion identification from the MS^n analysis of SAL $[(SAL-H+Na)+H]^+$.

Observed *m/z*	Formula	Theoretical *m/z*	Error (ppm)	Identification
MS/MS 773 - Figure 2(a)				
773.4805	$C_{42}H_{70}O_{11}Na^+$	773.4810	0.65	PI = $[(SAL-H+Na)+H]^+$
755.4702	$C_{42}H_{68}O_{10}Na^+$	755.4705	0.40	773 loss of H_2O
737.4598	$C_{42}H_{66}O_9Na^+$	737.4599	0.14	755 loss of H_2O
729.4911	$C_{41}H_{70}O_9Na^+$	729.4912	0.14	773 loss of CO_2
727.4753	$C_{41}H_{68}O_9Na^+$	727.4756	0.41	755 loss of CO
711.4802	$C_{41}H_{68}O_8Na^+$	711.4806	0.56	755 loss of CO_2
531.3287	$C_{29}H_{48}O_7Na^+$	531.3292	0.94	773 loss of $C_{13}H_{22}O_4$ (mass 242)
513.3181	$C_{29}H_{46}O_6Na^+$	513.3187	1.17	531 loss of H_2O
431.2399	$C_{23}H_{36}O_6Na^+$	431.2402	0.70	531 loss of $C_6H_{12}O$ (mass 100)
413.2295	$C_{23}H_{34}O_5Na^+$	413.2298	0.73	431 loss of H_2O
403.2452	$C_{22}H_{36}O_5Na^+$	403.2455	0.74	431 loss of CO
365.2296	$C_{19}H_{34}O_5Na^+$	365.2298	0.55	531 loss of $C_{10}H_{14}O_2$ (mass 166)
265.1409	$C_{13}H_{22}O_4Na^+$	265.1410	0.38	531 loss of $C_{16}H_{26}O_2$ (mass 266)<br>365 loss of $C_6H_{12}O$ (mass 100)
MS^2 773 > MS^3 531 - Figure 2(b)				
531.3285	$C_{29}H_{48}O_7Na^+$	531.3292	1.32	PI
513.2179	$C_{29}H_{46}O_6Na^+$	513.3187	1.56	531 loss of H_2O
503.3336	$C_{28}H_{48}O_6Na^+$	503.3343	1.39	531 loss of CO
487.3386	$C_{28}H_{48}O_5Na^+$	487.3394	1.64	531 loss of CO_2
475.3023	$C_{26}H_{44}O_6Na^+$	475.3030	1.47	531 loss of C_3H_4O
431.2397	$C_{23}H_{36}O_6Na^+$	431.2402	1.16	531 loss of $C_6H_{12}O$ (mass 100)
413.2294	$C_{23}H_{34}O_5Na^+$	413.2298	0.97	431 loss of H_2O
403.2451	$C_{22}H_{36}O_5Na^+$	403.2455	0.99	431 loss of CO
391.2087	$C_{20}H_{32}O_6Na^+$	391.2091	1.02	?
365.2294	$C_{19}H_{34}O_5Na^+$	365.2298	1.10	531 loss of $C_{10}H_{14}O_2$ (mass 166)
265.1407	$C_{13}H_{22}O_4Na^+$	265.1410	1.13	531 loss of $C_{16}H_{26}O_2$ (mass 266)<br>365 loss of $C_6H_{12}O$ (mass 100)
MS^2 773 > MS^3 431 – Figure 2(c)				
431.2398	$C_{23}H_{36}O_6Na^+$	431.2402	0.93	PI
413.2293	$C_{23}H_{34}O_5Na^+$	413.2298	1.21	431 loss of H_2O
403.2449	$C_{22}H_{36}O_5Na^+$	403.2455	1.49	431 loss of CO
387.2501	$C_{22}H_{36}O_4Na^+$	387.2506	1.29	431 loss of CO_2
369.2395	$C_{22}H_{34}O_3Na^+$	369.2400	1.35	387 loss of H_2O
361.1982	$C_{19}H_{30}O_5Na^+$	361.1985	0.83	431 loss of C_4H_6O
333.2032	$C_{18}H_{30}O_4Na^+$	333.2036	1.20	subfrag of 403
317.1356	$C_{16}H_{22}O_5Na^+$	317.1359	0.95	?
261.1094	$C_{13}H_{18}O_4Na^+$	261.1097	1.15	?
249.1458	$C_{13}H_{22}O_3Na^+$	249.1461	1.20	subfrag of 413
207.1352	$C_{11}H_{20}O_2Na^+$	207.1356	1.93	subfrag of 413
MS^2 773 > MS^3 755 – Figure S1				
755.4896	$C_{42}H_{68}O_{10}Na^+$	755.4705	1.19	PI
737.4591	$C_{42}H_{66}O_9Na^+$	737.4599	1.08	755 loss of H_2O
711.4798	$C_{41}H_{68}O_8Na^+$	711.4806	1.12	755 loss of CO_2
513.3178	$C_{29}H_{46}O_6Na^+$	513.3187	1.75	755 loss of $C_{13}H_{22}O_4$ (mass 242)
431.2397	$C_{23}H_{36}O_6Na^+$	431.2402	1.16	subfrag
413.2292	$C_{23}H_{34}O_5Na^+$	413.2298	1.45	431 loss of H_2O
MS^2 773 > MS^3 513 – Figure S2				
513.3178	$C_{29}H_{46}O_6Na^+$	513.3187	1.75	PI
457.2917	$C_{26}H_{42}O_5Na^+$	457.2924	1.53	513 loss of C_3H_4O (mass 56)
413.2291	$C_{23}H_{34}O_5Na^+$	413.2298	1.69	513 loss of $C_6H_{12}O$ (mass 100)
373.1979	$C_{20}H_{30}O_5Na^+$	373.1985	1.61	513 loss of $C_9H_{16}O$ (mass 140)

Table S2. Contd.

Observed *m/z*	Formula	Theoretical *m/z*	Error (ppm)	Identification
MS^2 773 > MS^3 531 > MS^4 431 – Figure S3				
431.2397	$C_{23}H_{36}O_6Na^+$	431.2402	1.16	PI
413.2293	$C_{23}H_{34}O_5Na^+$	413.2298	1.21	431 loss of H_2O
403.2449	$C_{22}H_{36}O_5Na^+$	403.2455	1.49	431 loss of CO
387.2501	$C_{22}H_{36}O_4Na^+$	387.2506	1.29	431 loss of CO_2
369.2395	$C_{22}H_{34}O_3Na^+$	369.2400	1.35	387 loss of H_2O
361.1981	$C_{19}H_{30}O_5Na^+$	361.1985	1.11	431 loss of C_4H_6O
333.2033	$C_{18}H_{30}O_4Na^+$	333.2036	0.90	subfrag of 403
265.1407	$C_{13}H_{22}O_4Na^+$	265.1410	1.13	subfrag of 431
249.1457	$C_{13}H_{22}O_3Na^+$	249.1461	1.61	subfrag of 431
207.1352	$C_{11}H_{20}O_2Na^+$	207.1356	1.93	subfrag of 265
MS^2 773 > MS^3 413 – Figure S4				
413.2292	$C_{23}H_{34}O_5Na^+$	413.2298	1.45	PI
395.2187	$C_{23}H_{32}O_4Na^+$	395.2193	1.52	413 loss of H_2O
385.2345	$C_{22}H_{34}O_4Na^+$	385.2349	1.04	413 loss of CO
355.1876	$C_{20}H_{28}O_4Na^+$	355.1880	1.13	413 loss of C_3H_6O
343.1876	$C_{19}H_{28}O_4Na^+$	343.1880	1.17	385 loss of C_3H_6
327.1565	$C_{18}H_{24}O_4Na^+$	327.1567	0.61	subfrag
311.1614	$C_{18}H_{24}O_3Na^+$	311.1618	1.29	subfrag
299.1249	$C_{16}H_{20}O_4Na^+$	299.1254	1.67	395 loss of
271.1301	$C_{15}H_{20}O_3Na^+$	271.1305	1.48	299 loss of CO
243.0988	$C_{13}H_{16}O_3Na^+$	243.0992	1.65	271 loss of C_2H_4
MS^2 773 > MS^3 431 > MS^4 413 – Figure S5				
413.2293	$C_{23}H_{34}O_5Na^+$	413.2298	1.21	PI
395.2190	$C_{23}H_{32}O_4Na^+$	395.2193	0.76	413 loss of H_2O
385.2345	$C_{22}H_{34}O_4Na^+$	385.2349	1.56	413 loss of CO
369.2396	$C_{22}H_{34}O_3Na^+$	369.2400	1.08	subfrag
355.1877	$C_{20}H_{28}O_4Na^+$	355.1880	0.84	subfrag
343.1876	$C_{19}H_{28}O_4Na^+$	343.1880	1.17	subfrag
317.1357	$C_{16}H_{22}O_5Na^+$	317.1259	0.63	subfrag
287.1616	$C_{16}H_{24}O_3Na^+$	287.1618	0.70	subfrag
243.0988	$C_{13}H_{16}O_3Na^+$	243.0992	1.65	subfrag
MS^2 773 > MS^3 331 > MS^4 365 – Figure S6				
365.2294	$C_{19}H_{34}O_5Na^+$	365.2298	1.10	PI
265.1408	$C_{13}H_{22}O_4Na^+$	265.1410	0.75	365 loss of $C_6H_{12}O$ (mass 100)
MS^2 773 > MS^3 531 > MS^4 265 – Figure S7				
265.1408	$C_{13}H_{22}O_4Na^+$	265.1410	0.75	PI
221.1509	$C_{12}H_{22}O_2Na^+$	221.1512	1.36	265 loss of CO_2
207.0989	$C_{10}H_{16}O_3Na^+$	207.0992	1.45	265 loss of C_3H_6O

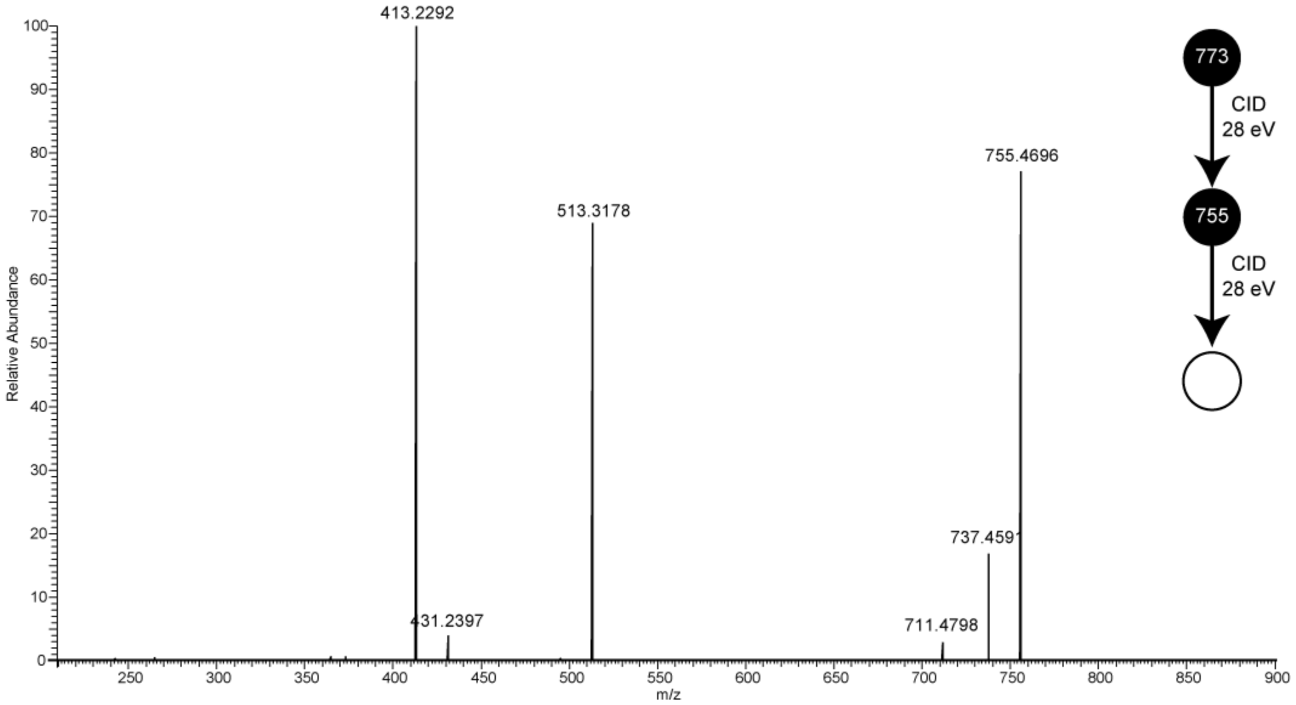

Figure S1. Positive ion ESI-CID-MS^3 spectrum of $[(SAL\text{-}H+Na)+H]^+$ (PI *m/z* 755).

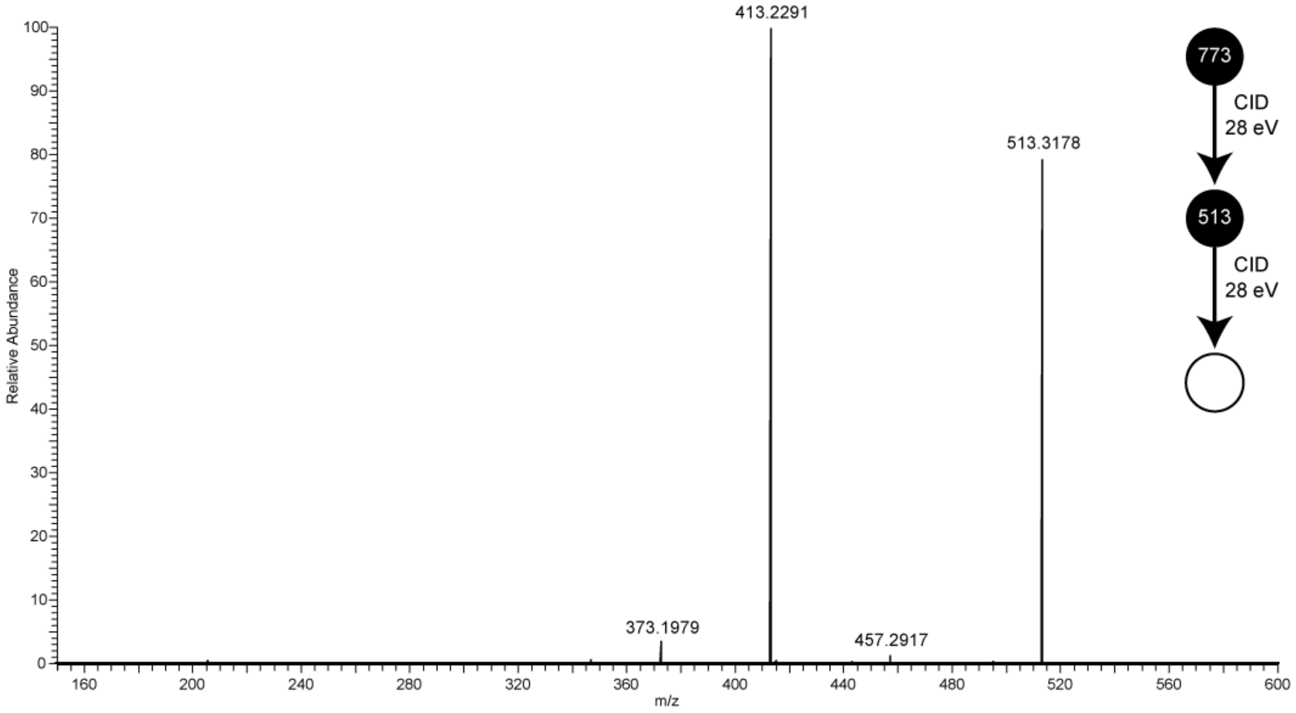

Figure S2. Positive ion ESI-CID-MS^3 spectrum of $[(SAL\text{-}H+Na)+H]^+$ (PI *m/z* 513).

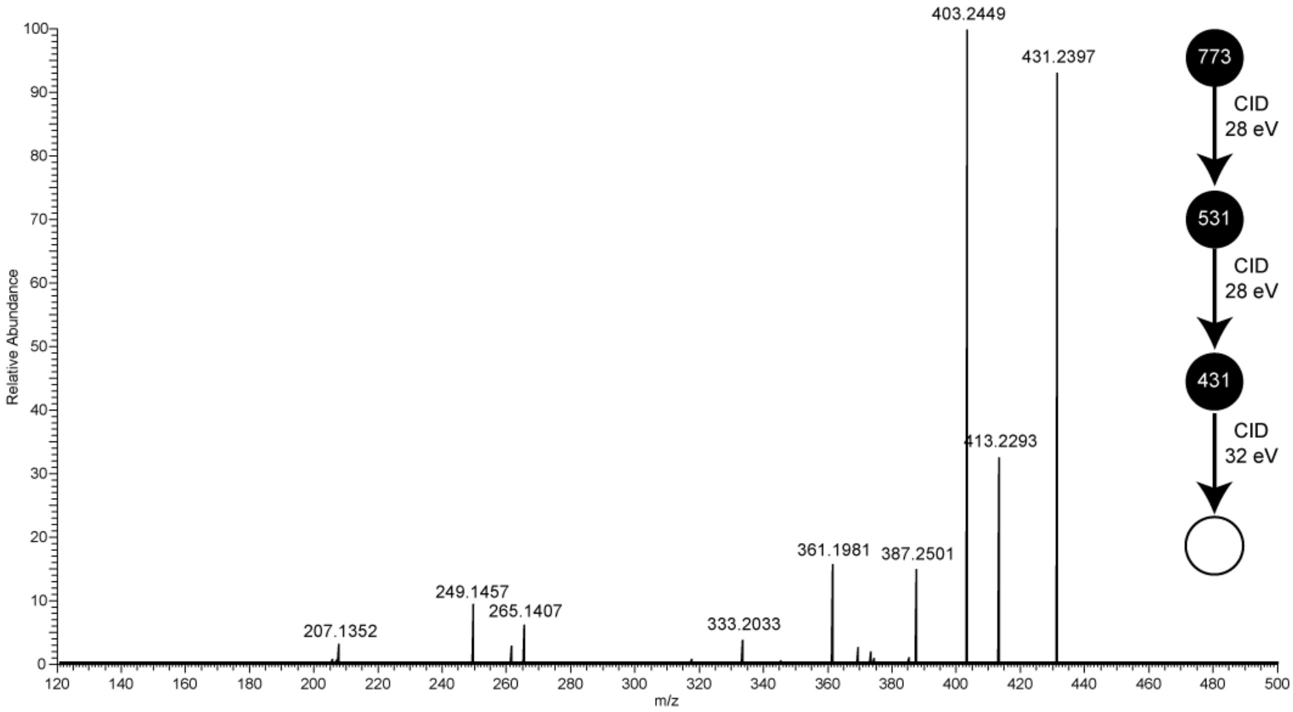

Figure S3. Positive ion ESI-CID-MS^4 spectrum of $[(SAL\text{-}H+Na)+H]^+$ (PI *m/z* 431).

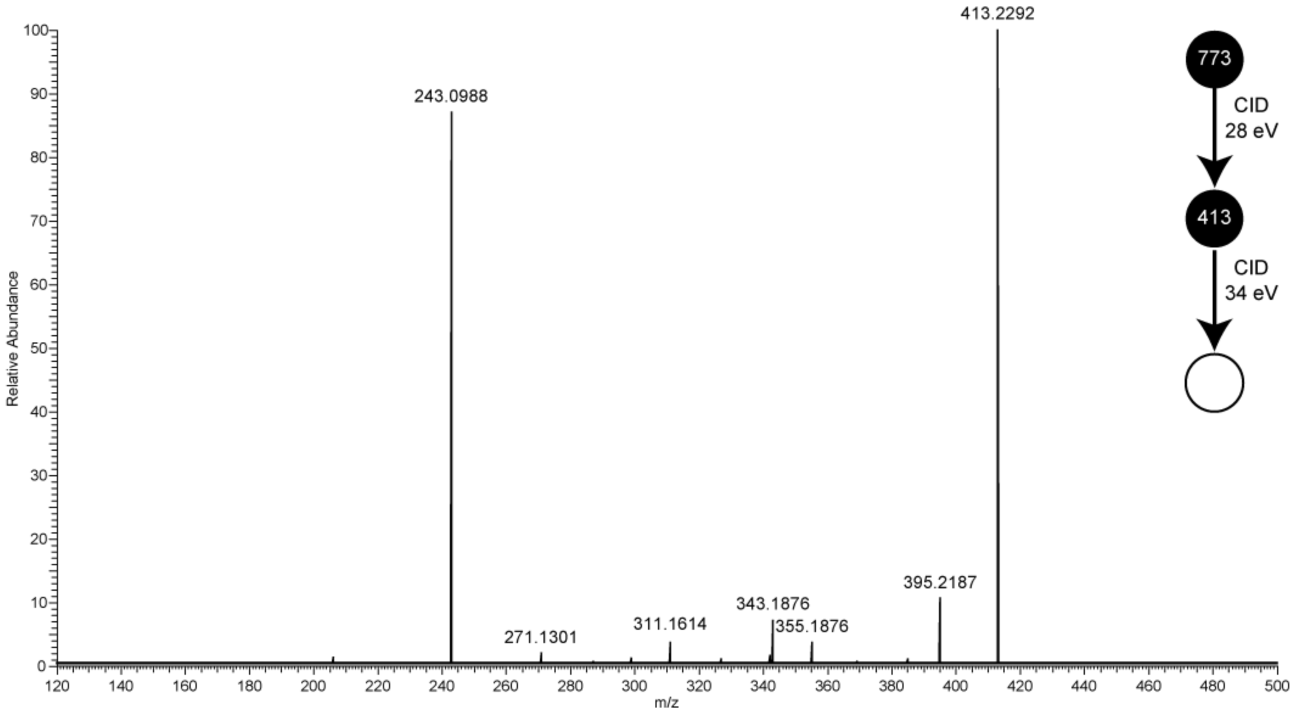

Figure S4. Positive ion ESI-CID-MS^3 spectrum of $[(SAL\text{-}H+Na)+H]^+$ (PI *m/z* 413).

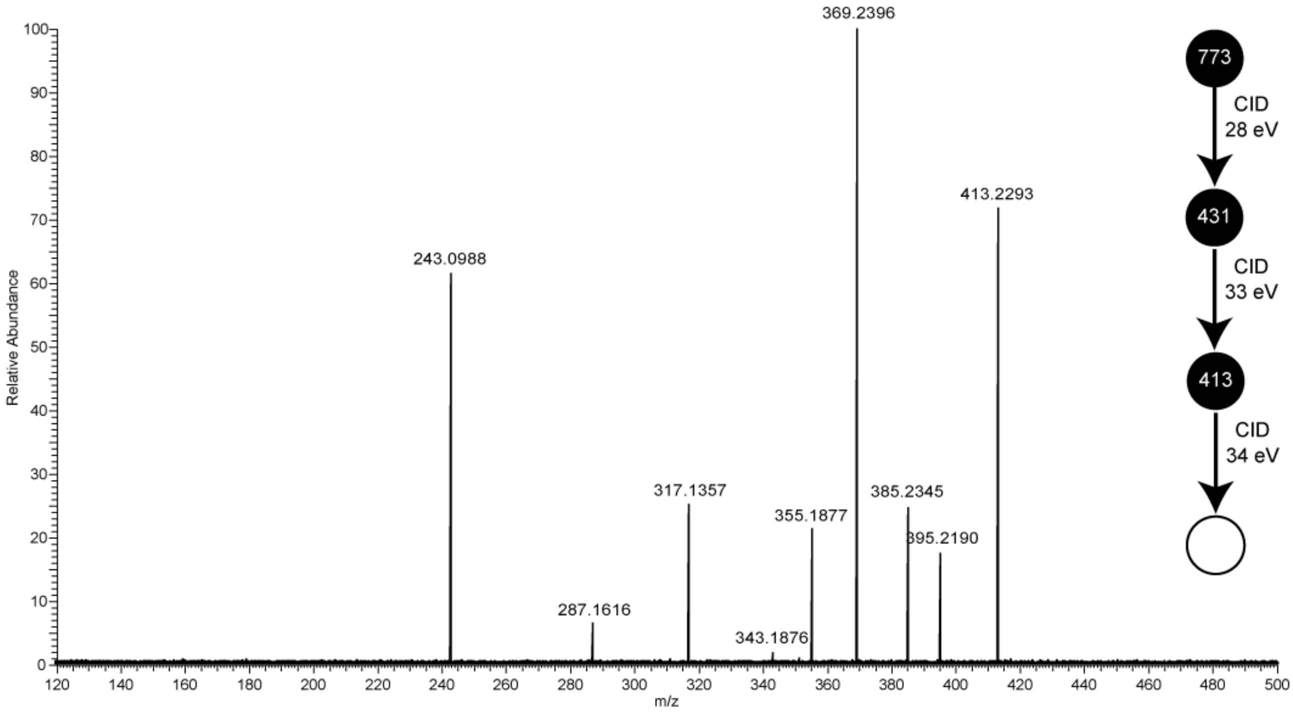

Figure S5. Positive ion ESI-CID-MS^4 spectrum of $[(SAL\text{-}H+Na)+H]^+$ (PI *m/z* 413).

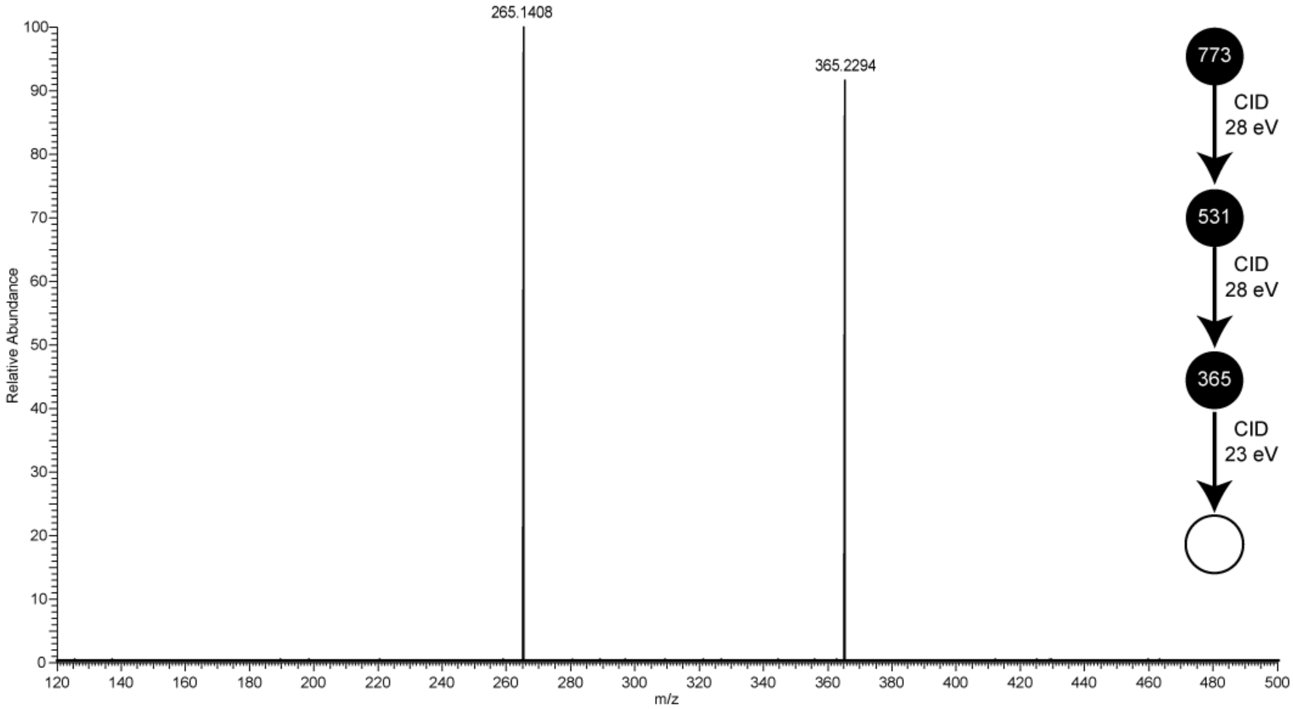

Figure S6. Positive ion ESI-CID-MS^4 spectrum of $[(SAL\text{-}H+Na)+H]^+$ (PI *m/z* 365).

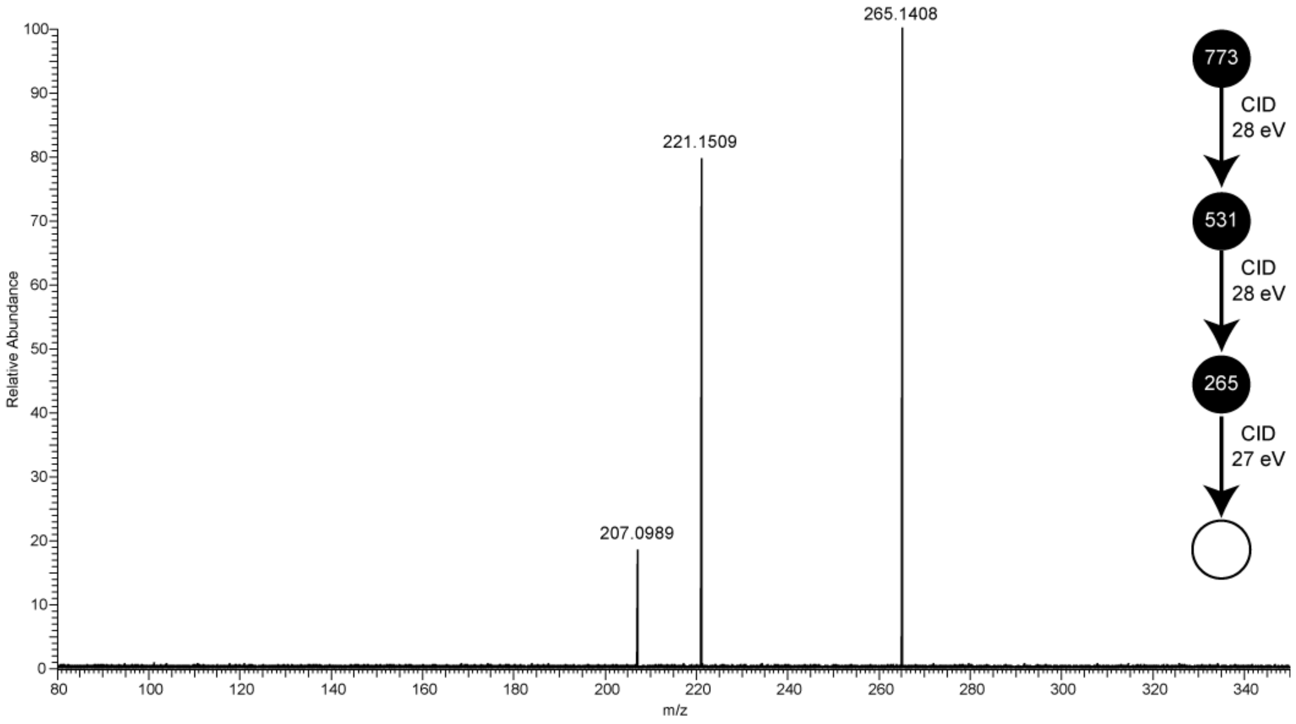

Figure S7. Positive ion ESI-CID-MS^4 spectrum of $[(SAL-H+Na)+H]^+$ (PI *m/z* 265).
